# A Pediatric Case of Spinal Intradural Epidermoid Cyst: A Rare Encounter

**DOI:** 10.7759/cureus.49970

**Published:** 2023-12-05

**Authors:** Fahad N Alsulami, Fahad M Alotaibi, Layan S Alfraidi, Nasser F Alotaibi, Razan E Alnami, Mohammed F Alotaibi, Reema M Musllet, Ahlam Alharbi

**Affiliations:** 1 General Practice, Dar Al Uloom University, Riyadh, SAU; 2 Dentistry, Imam Abdulrahman Bin Faisal University, Dammam, SAU; 3 General Practice, Princess Nourah Bint Abdul Rahman University, Riyadh, SAU; 4 Family Medicine, Primary Health Care Center, Riyadh, SAU

**Keywords:** intradural epidermoid cyst, spinal tumors, neurological deficits, chemical meningitis, radicular pain

## Abstract

Intradural epidermoid cysts of the spine are rare congenital lesions. Their etiology is thought to stem from ectodermal remnants during embryonic development. They result in a diverse clinical presentation, often marked by an insidious onset and variable neurological deficits. Timely diagnosis is crucial for optimizing patient outcomes. We present the case of a 10-year-old male child presenting a six-month history of worsening back pain, intermittent leg weakness, and urinary incontinence. The physical examination revealed tenderness over the lower thoracic and lumbar spine, lower limb weakness, hyperreflexia, and sensory deficits. The diagnostic work-up, including cerebrospinal fluid analysis and magnetic resonance imaging, confirmed the presence of an intradural epidermoid cyst in the lumbosacral region. Surgical excision resulted in complete resection, with subsequent improvement in neurological deficits. This pediatric case underscores the importance of maintaining a high index of suspicion for unexplained neurological deficits. Characteristic imaging findings played a pivotal role in the diagnosis, guiding successful surgical intervention and achieving favorable outcomes.

## Introduction

Intradural epidermoid cysts of the spine are rare congenital lesions characterized by the presence of stratified squamous epithelium and keratin debris within the spinal canal. These cysts account for less than 1% of all spinal tumors, making them an uncommon yet clinically significant entity [1]. While their etiology remains unclear, current literature suggests that these cysts arise from ectodermal remnants during embryonic development. Clinical manifestations of spinal intradural epidermoid cysts are diverse, often presenting insidiously with symptoms such as back pain, radicular pain, and motor or sensory deficits. The compressive effects on adjacent neural structures, coupled with the potential for chemical meningitis due to cyst contents released into the cerebrospinal fluid, contribute to the variable clinical phenotype [1]. Timely diagnosis and intervention are imperative for optimizing patient outcomes. We present the case of a previously healthy child with an intradural spinal epidermoid cyst in the lumbosacral region.

## Case presentation

A 10-year-old male child presented to the pediatric neurosurgery clinic with a six-month history of progressively worsening back pain, intermittent leg weakness, and occasional urinary incontinence. There was no history of fecal incontinence. The patient's parents reported no history of trauma, febrile illnesses, or other significant medical conditions. Additionally, there was no family history of neurologic disorders or congenital anomalies. The child's developmental milestones were age-appropriate, and he had not experienced any recent growth spurts or weight changes.

Upon thorough physical examination, the patient exhibited tenderness localized to the lower thoracic and lumbar spine, without any apparent signs of deformity. The neurological assessment revealed subtle weakness in specific musculature groups of the lower extremities, notably affecting the left quadriceps and ankle dorsiflexors. This weakness was accompanied by hyperreflexia and a positive Babinski sign. The sensory examination demonstrated diminished pinprick sensation in a well-defined pattern, specifically in the left L4 to S1 dermatomes, when compared to the right side. Importantly, the cranial nerve examination yielded normal results, and there were no discernible indications of cerebellar dysfunction.

Given the concerning neurological findings, a comprehensive work-up was initiated. Routine laboratory investigations, including complete blood count, electrolytes, and inflammatory markers, were all within normal limits. Further diagnostic procedures included lumbar puncture (performed at the level of L3), which showed an elevated protein level in the cerebrospinal fluid without pleocytosis. The differential diagnoses considered included other spinal cord tumors, infectious etiologies, and vascular malformations.

**Figure 1:**
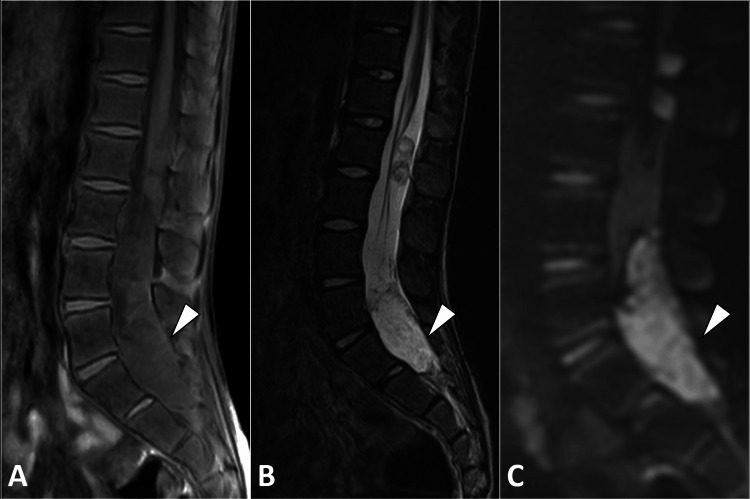
Sagittal MR images of lumbosacral spines illustrate an intradural lesion (arrowhead) marked by distinct features: low-intensity signal on T1-weighted image (A), high-intensity signal on T2-weighted image (B), and notable diffusion restriction (C). These findings are indicative of an epidermoid cyst. MR: Magnetic resonance.

Magnetic resonance imaging of the spine was performed, which revealed well-defined intradural lesions at the level of the conus medullaris and the lumbosacral junction. The lesions appeared hypointense on T1-weighted images and hyperintense on T2-weighted images and showed restricted diffusion, consistent with an epidermoid cyst. The lesion was compressing and displacing nerve roots. These findings were characteristic of intradural epidermoid cysts (Figure 1).

**Figure 2:**
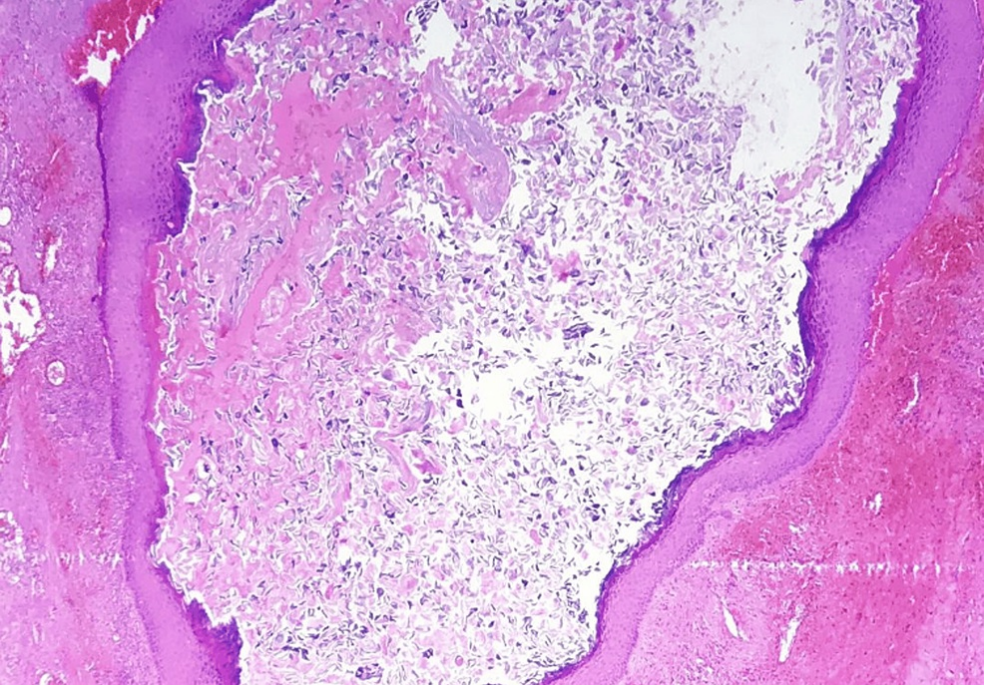
Histopathological examination of the excised intradural epidermoid cyst reveals stratified squamous epithelium and keratin debris characteristic of epidermoid cysts.

The patient underwent surgical excision of the cyst, and intraoperatively, the lesions were found to be well-encapsulated and adherent to the spinal cord. Gross total resection of both lesions was achieved using laminectomy procedure, and histopathological examination confirmed the diagnosis of an epidermoid cyst (Figure 2). Postoperatively, the patient showed gradual improvement in motor strength, and urinary incontinence was resolved. Rehabilitation therapy was initiated to address residual weakness and optimize functional outcomes.

The hospital course was uneventful, with the patient being discharged on postoperative day five. Follow-up evaluations at one month, three months, and six months postoperatively demonstrated sustained neurological improvement, and the patient resumed normal activities without any recurrence of symptoms.

## Discussion

The presented case of a spinal intradural epidermoid cyst in a pediatric patient underscores the rarity of this entity and the diagnostic challenges it poses. The clinical presentation, characterized by insidious onset of back pain, intermittent leg weakness, and urinary incontinence, aligns with the diverse spectrum of symptoms reported in the literature. The non-specific nature of these symptoms often leads to delays in diagnosis, emphasizing the importance of maintaining a high index of suspicion for spinal intradural epidermoid cysts in the presence of unexplained neurological deficits [1,2].

The characteristic imaging findings on MRI, demonstrating T1 hypointensity, T2 hyperintensity, and restriction of diffusion, were key in the diagnosis in this case. The utility of MRI in preoperative planning and the delineation of anatomical relationships cannot be overstated, aligning with previous reports that highlight its role as the imaging modality of choice for spinal intradural epidermoid cysts [2,3].

Surgical intervention, in the form of gross total resection, remains the cornerstone of management for spinal intradural epidermoid cysts. The encapsulated nature of these lesions, as observed intraoperatively in our case, enables surgical excision with favorable outcomes. While complete resection is associated with a low risk of recurrence, the potential for intraoperative rupture and spillage of cyst contents must be carefully managed to minimize the risk of chemical meningitis. In the pediatric population, careful attention to preserving neurological function is paramount, given the potential for long-term sequelae [2-4].

The postoperative course in our patient demonstrated gradual neurological improvement, aligning with the reported outcomes in the limited existing literature [2,5]. The resolution of urinary incontinence and the restoration of motor strength highlight the potential for favorable outcomes with timely and appropriate surgical intervention. Long-term follow-up will be essential to monitor for any recurrence or the development of complications [1,5].

## Conclusions

In conclusion, the presented case of a spinal intradural epidermoid cyst in a pediatric patient highlights the diagnostic intricacies and therapeutic considerations associated with this rare pathology. Timely recognition facilitated by characteristic imaging findings and judicious surgical intervention resulted in a favorable outcome with neurological improvement. This case underscores the importance of maintaining awareness of spinal intradural epidermoid cysts as a potential cause of unexplained neurological deficits, particularly in the pediatric population. Continued reporting of individual cases and collaborative research efforts will contribute to the refinement of diagnostic and therapeutic strategies for this uncommon but clinically significant condition.
